# Investigating a hybrid extreme learning machine coupled with Dingo Optimization Algorithm for modeling liquefaction triggering in sand-silt mixtures

**DOI:** 10.1038/s41598-024-61059-6

**Published:** 2024-05-11

**Authors:** Mohammed Majeed Hameed, Adil Masood, Aman Srivastava, Norinah Abd Rahman, Siti Fatin Mohd Razali, Ali Salem, Ahmed Elbeltagi

**Affiliations:** 1 Department of Civil Engineering, Al-Maarif University College, Ramadi, Iraq; 2 Department of Computer Science, Al-Maarif University College, Ramadi, Iraq; 3 Department of Natural and Applied Sciences, TERI School of Advanced Studies, New Delhi, India; 4 Department of Civil Engineering, Indian Institute of Technology (IIT) Kharagpur, Kharagpur, West Bengal 721302 India; 5 Department of Civil Engineering, Faculty of Engineering and Built Environment, Universiti Kebangsaan Malaysia, 43600 Bangi, Selangor Malaysia; 6 Smart and Sustainable Township Research Centre (SUTRA), Universiti Kebangsaan Malaysia (UKM), 43600 Bangi, Selangor Malaysia; 7 Civil Engineering Department, Faculty of Engineering, Minia University, Minia, 61111 Egypt; 8 Structural Diagnostics and Analysis Research Group, Faculty of Engineering and Information Technology, University of Pécs, Pécs, Hungary; 9 Agricultural Engineering Department, Faculty of Agriculture, Mansoura University, Mansoura, 35516 Egypt

**Keywords:** Liquefaction, Earthquake, Dingo Optimization Algorithm, Non-linear normalization, Natural hazards, Solid Earth sciences, Engineering

## Abstract

Liquefaction is a devastating consequence of earthquakes that occurs in loose, saturated soil deposits, resulting in catastrophic ground failure. Accurate prediction of such geotechnical parameter is crucial for mitigating hazards, assessing risks, and advancing geotechnical engineering. This study introduces a novel predictive model that combines Extreme Learning Machine (ELM) with Dingo Optimization Algorithm (DOA) to estimate strain energy-based liquefaction resistance. The hybrid model (ELM-DOA) is compared with the classical ELM, Adaptive Neuro-Fuzzy Inference System with Fuzzy C-Means (ANFIS-FCM model), and Sub-clustering (ANFIS-Sub model). Also, two data pre-processing scenarios are employed, namely traditional linear and non-linear normalization. The results demonstrate that non-linear normalization significantly enhances the prediction performance of all models by approximately 25% compared to linear normalization. Furthermore, the ELM-DOA model achieves the most accurate predictions, exhibiting the lowest root mean square error (484.286 J/m^3^), mean absolute percentage error (24.900%), mean absolute error (404.416 J/m^3^), and the highest correlation of determination (0.935). Additionally, a Graphical User Interface (GUI) has been developed, specifically tailored for the ELM-DOA model, to assist engineers and researchers in maximizing the utilization of this predictive model. The GUI provides a user-friendly platform for easy input of data and accessing the model's predictions, enhancing its practical applicability. Overall, the results strongly support the proposed hybrid model with GUI serving as an effective tool for assessing soil liquefaction resistance in geotechnical engineering, aiding in predicting and mitigating liquefaction hazards.

## Introduction

The concept of liquefaction in soil science revolves around the phenomenon wherein soil undergoes a loss of strength and stiffness, adopting liquid-like properties.. This usually occurs during an earthquake or other seismic activities when a sudden increase in pore water pressure within the soil causes it to lose its ability to support dynamic loading. When liquefaction happens, the ground essentially turns into a fluid-like state, leading to the sinking, tilting, or sliding of structures built on the affected soil. Soil liquefaction increases the intensity of the damage caused by earthquakes. Assessment of the various literature surveys on liquefaction-induced devastation reveals five significant occurrences, namely the earthquakes in Niigata in 1964, Alaska in 1964, Kobe in 1995, Christchurch in 2011, and Sulawesi in 2018^1–5^. Under the initial stress conditions, seismic waves generated during an earthquake cause the ground to shake, subjecting the soil to dynamic loading in the form of cyclic shear stress. This cyclic loading rearranges soil particles, increasing pore water pressure within the soil due to the inability of water to escape quickly. As pore water pressure rises, effective stress that holds the soil particles together, gradually decreases until it reaches zero or becomes negative, causing the soil to lose its cohesion and behave like a fluid. Structures atop liquified soil may sink or tilt, resulting in significant damage. After the seismic event ceases, excess pore water pressure gradually dissipates, but the damage remains. The occurrence of liquefaction is contingent upon several factors within the soil, including its composition, density, saturation level and intensity of seismic shaking^6,7^.

To mitigate the potential loss of life and property, it is crucial to conduct a liquefaction hazard analysis at sites vulnerable to this phenomenon as a fundamental step in seismic risk management^8^. Numerous studies have been conducted to improve the precision of liquefaction prediction methods; discussed here. The stress-based approach is a widely used method for assessing liquefaction potential. It determines the soil's likelihood of liquefying by examining its stress state during seismic loading.It estimates the cyclic shear stress imposed on the soil and compares it with the soil's resistance, often considering factors such as shear stress history and soil properties^9–11^. The energy-based approach focuses on the energy imparted by seismic waves and compares it with the energy dissipated by the soil during cyclic loading. This method considers factors such as stress–strain hysteresis loops to assess liquefaction susceptibility^12–14^. There are techniques like the empirical methods, which rely on observed correlations between liquefaction and factors such as standard penetration test (SPT) N-values or shear wave velocity^15–17^. Empirical methods often offer practical solutions based on historical data but may lack precision in diverse geological settings.

Numerical modeling techniques, such as finite element analysis and discrete element modeling, enable detailed simulations of soil behavior, accounting for complex factors like soil heterogeneity and three-dimensional stress states^18,19^. However, the phenomenon of liquefaction is inherently nonlinear, and the existing empirical and statistical models found in the literature have limitations in accurately predicting and achieving desirable outcomes. Additionally, certain approaches, such as model-based finite element methods, often rely on numerous assumptions and extensive data requirements. To enhance prediction accuracy and flexibility, Machine Learning (ML) models can offer a viable alternative to the existing techniques. This is primarily attributed to the capability of ML models to identify intricate relationships between input features and output^20,21^. Also, ML models can effectively capture complex patterns and dependencies, allowing them to adapt and learn from the data, ultimately leading to improved prediction accuracy and a more flexible approach in various domains^22–24^.

In the field of liquefaction prediction, various ML techniques have recently seen a surge in adoption for assessing liquefaction potential^25^. Some researchers have developed support vector regression (SVR) and extreme gradient boosting (XGBoost) based models to predict liquefaction using various soil and seismic^26,27^. These studies have demonstrated promising results in terms of predictive accuracy. Moreover, the use of artificial neural network (ANN) prediction-based models, and adaptive neuro-fuzzy inference system (ANFIS) models have been implemented to forecast soil liquefaction, resulting in improved prediction accuracy^28–30^. Other researchers have investigated the potential of extreme learning machines (ELM), a modified version of ANN, for predicting soil liquefaction^31^. Their findings indicated that ELM exhibited a higher prediction capacity, achieving an accuracy of 87.5. Given the strong predictive performance of classical ML models, the challenge lies in finding the optimal hyperparameters that significantly impact model predictions. To address this, geotechnical researchers have turned to various metaheuristic algorithms, including genetic algorithm (GA), grey wolf optimization (GWO), and differential evolution (DE)^32,33^. By incorporating these algorithms into hybrid models, researchers have observed a substantial improvement in prediction accuracy^34^.

In recent years, the integration of machine learning techniques into geotechnical engineering has shown promising potential for addressing complex soil mechanics phenomena. A recent study has explored the application of ANN based models for estimating geotechnical properties of lime-stabilised clayey soils, demonstrating the versatility of machine learning approaches in geotechnical engineering-based problems^35^. Other researchers have investigated machine learning methods for predicting the maximum dry density of soil samples^36^. It was observed that the constructed models exhibited significant generalization capability when predicting correlations among soil properties. Besides, other scholars have employed various state-of-the-art modeling techniques for estimating the ground liquefaction potential, highlighting the utility of ML approaches in framing soil liquefaction decisions under given conditions^37^. Moreover, some investigations have explored the role of tunnel boring machine (TBM) tail-grouting on surface settlement in coarse-grained soils, emphasizing the importance of numerical modeling and field testing in geotechnical engineering practices^38^. In addition, several researchers demonstrated the effectiveness of supervised learning methods in modeling unconfined compressive strength prediction of stabilized soils, showcasing the potential of ML techniques in simulating geotechnical properties under real conditions^39^. Moreover, other studies have predicted the potential for earthquake-induced liquefaction in fine-grained soil with minimal uncertainty and human intervention, showcasing the effectiveness of machine learning methods in evaluating liquefaction prediction performance for practical earthquake engineering applications^40,41^. These studies collectively illustrate the wide-ranging applicability of ML methods in geotechnical engineering domain, motivating further exploration of ML-based approaches for tackling soil liquefaction prediction challenges.

According to the available research^42^, there is a recognized shortage of studies in the prediction of soil liquefaction potential within the field of geological/geotechnical engineering^43^. Consequently, in the present study, liquefaction potential was assessed using a ML-based approach which integrated the Extreme Learning Machine (ELM) with a novel meta-heuristic algorithm referred to as the Dingo Optimization Algorithm (DOA). The DOA played a crucial role in optimizing the proposed hybrid model (ELM-DOA), resulting in improved performance and efficacy^44^. 

The study further explores the application of non-linear normalization technique and compares the accuracy of ML models using this technique with the commonly used linear normalization approach. Furthermore, the prediction accuracy of the ELM-DOA model has been compared with classic ELM, ANFIS, and other ML models. The research also aims to investigate the use of non-linear normalization in ML model development and evaluate its predictive accuracy compared to the widely used linear normalization method. It seeks to highlight the potential advantages and effectiveness of non-linear normalization techniques in improving ML model performance. Finally, the study introduces a graphical user interface (GUI) tailored to aid engineers and researchers in effectively utilizing the developed model.

## Methods and materials

### Data collection

The energy dissipation theories were first introduced by^45^ who provided an understanding of the pore water pressure generation inside the soil skeleton. The dissipated energy (W) required to trigger liquefaction in a soil volume can be obtained from undrained cyclic triaxial tests, which are highly dependent on effective confining pressure. In terms of the silt-sand mixture, several parameters taken into consideration for predicting soil composition are relative density (Dr), uniformity coefficient (Cu), mean grain size (D50), initial mean effective confining pressure (sigma c), and percentage of fine content (FC). The database adopted in this study is based on laboratory tests conducted during previous studies^46–49^. Including these parameters is essential for predicting soil composition and liquefaction resistance in sand-silt mixtures. Recent research provides insights into identifying and benchmarking significant factors related to soil liquefaction, further supporting the discussion on the selection of input parameters^50^. For instance, relative density (Dr) reflects the compactness of soil particles, which directly influences soil strength and susceptibility to liquefaction. Similarly, parameters such as uniformity coefficient (Cu) and mean grain size (D50) offer insights into soil gradation and particle size distribution, affecting soil stability and deformation characteristics during cyclic loading. Initial mean effective confining pressure (sigma c) plays a crucial role in confining soil particles and controlling pore water pressure generation, while the percentage of fine content (FC) influences soil cohesion and internal friction angle, impacting soil liquefaction potential.

The dataset used in this study comprises 144 samples (see Tables S-1 and S-2), which were utilized to train the energy-based liquefaction resistance of sand-silt mixtures. Out of these samples, 100 were allocated for training the models, while the remaining samples were used for testing purposes. In this study, the data was divided into training and testing sets randomly, following a widely used approach in previous works^51–55^ to reduce bias between the two sets. To ensure repeatability in the data division process, a specific random seed value was employed and recorded. This allows for the exact replication of the division, ensuring consistent results. After this step, the training and testing data were stored and used for training the ML models. It is worth noting that the overall data, including the training and testing sets, are provided in the ESM Appendix. Importantly, there is no repeated data in the dataset, as each observation is uniquely assigned to either the training or testing set. This ensures the integrity and validity of the data used for model training and evaluation.

The statistics in Table 1 include the maximum (*X*_*max*_), average (*X*_*av*_), minimum (*X*_*min*_), standard deviation (*X*_*st.d*_), skewness (*S*_*sk*s_), and median (*X*_*med*_) values for each parameter. Table 1 shows that the statistical values (mean, maximum, median, st. dev, etc.) of the predictors (e.g., D50, Cu, Dr, FC, and Sigma c) in the training dataset are very similar to those in the testing dataset. Regarding liquefaction resistance (W), the average value in the training dataset is slightly higher than in the testing dataset by approximately 12%. However, the minimum value in the training dataset is lower than the corresponding value in the testing dataset by approximately 3.430%. These findings indicate a minor variation in the specific variable but do not suggest any significant bias in the overall data division procedure. Furthermore, the dataset has an overall maximum capacity energy of 15,000 J/m^3^, an average capacity energy of 2283.021 J/m^3^, and a minimum capacity energy of 385 J/m^3^. Overall, such statistics offer a synopsis of the dataset, providing an understanding of the range, distribution, and central tendency of various parameters pertaining to the energy-based liquefaction resistance of sand-silt mixtures.

**Table 1:** Statistical analysis of the experimental data.

Category	Statistics	Sigma c (Kp)	Dr (%)	FC (%)	Cu	D50 (mm)	W (J/m^3^)
Training samples	X_max_	400	93	60	28.120	0.260	12,230
	X_av_	103.470	62.807	14.710	8.101	0.165	2372.130
	X_min_	14.130	9.380	0	1.520	0.029	385
	X_st.d_	83.872	15.641	17.544	9.655	0.056	2156.920
	S_sks_	2.531	− 0.657	0.997	1.349	− 0.403	2.285
	X_med_	100	64.595	5	2.270	0.15	1705.450
Testing Samples	X_max_	400	88.020	60	28.120	0.26	15,000
	X_av_	92.13	60.505	13.627	6.277	0.155	2075.800
	X_min_	28.400	25	0	1.520	0.029	398.200
	X_st.d_	75.647	13.078	17.239	8.293	0.049	2545.110
	S_sks_	3.129	− 0.572	0.989	2.004	0.067	3.452
	X_med_	82.740	62	5	2.270	0.15	1317
Overall samples	X_max_	400	93	60	28.120	0.26	15,000
	X_av_	100.060	62.114	14.384	7.552	0.162	2283.02
	X_min_	14.130	9.380	0	1.520	0.029	385
	X_st.d_	81.389	14.909	17.4	9.275	0.054	2275.520
	S_sks_	2.6837	− 0.609	0.996	1.515	− 0.268	2.744
	X_med_	100	62.700	5	2.27	0.15	1609

### Extreme learning machine

**Figure 1:**
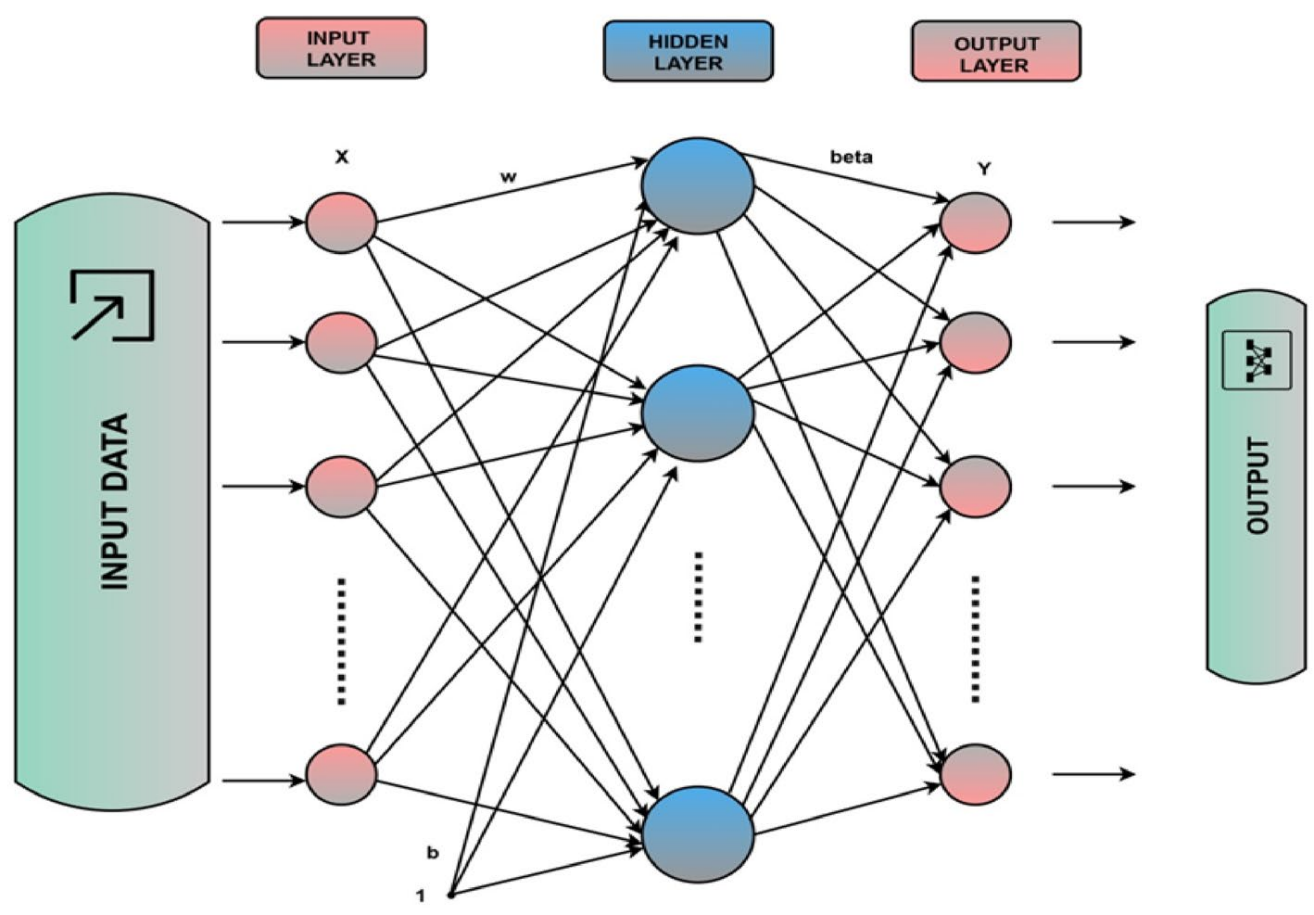
The basic structure of the ELM model.

The Extreme Learning Machine (ELM) stands out in machine learning for its simplicity and efficiency. Positioned within the category of feedforward neural networks, ELM distinguishes itself through a single-layer architecture, a departure from the conventional multi-layer structures found in traditional neural networks^56^. Notably, the connections between input and hidden layer neurons are randomly initialized^20,57^, contributing to the algorithm's adaptive nature. The main structure of the ELM can be illustrated in Fig. 1^58^.

ELM's learning process is marked by its remarkable speed, which makes it particularly adept at handling large datasets. The weights calculation, crucial for the model's performance, is achieved through a single learning iteration, eliminating the need for iterative optimization^59^. Despite its streamlined approach, ELM exhibits robust generalization capabilities, allowing it to navigate complex patterns within data effectively^58,60,61^. Equations (1–3) provide a breakdown of the equations associated with the ELM. Each neuron's output in the hidden layer is determined by the weighted sum of the input data (*w*_*i*_* x*) plus a bias term (*b*_*i*_), passed through an activation function *g*, as shown in Eq. (1). Here, *H*_*i*_ represents the output of the hidden neuron *i*; *g* is the activation function, typically a sigmoid, Gaussian, or ReLU function; w_*i*_ denotes the input weights connected to neuron *I*; *x* is the input data vector; *b*_*i*_ represents the bias term for neuron *i*.

1 $${H}_{i}=g({w}_{i} x+{b}_{i})$$

The output layer weights are calculated by performing a pseudo-inverse operation (*H*^+^) on the hidden layer output matrix (*H*) multiplied by the target output matrix (*T*), as shown in Eq. (2)^62^. Here, *Y* represents the predicted output matrix; *H* is the hidden layer output matrix obtained from the input data; *beta* denotes the output layer weights. The final predicted output (*Y*) is obtained by multiplying the hidden layer output matrix (*H*) with the calculated output layer weights (*beta*), as shown in Eq. (3)^63^.

2 $$beta={H}^{+}\times T$$

3 $$Y=H\times beta$$

These equations form the core of an ELM's functioning, where the network learns the mapping from input data to output by initializing input weights randomly and calculating output weights analytically without iterative optimization^64^. This approach results in fast learning and prediction capabilities in ELM. The suitability of ELM for liquefaction resistance prediction in sand-silt mixtures becomes evident in its ability to capture intricate relationships within the dataset. This adaptability extends to various data types, including geological data, aligning well with the complexity inherent in liquefaction resistance prediction. In the context of the hybrid model, ELM serves as the foundational machine learning algorithm, and its integration with the Dingo Optimization Algorithm (DOA) elevates its predictive accuracy. With a proven track record in various fields, ELM demonstrates versatility and reliability, although it is essential to note its limitations in interpretability, particularly when compared to more complex models^31,56,60,65,66^. In summary, the integration of ELM in the hybrid model underscores its efficiency, rapid learning capabilities, and adaptability to the intricacies of liquefaction resistance prediction.

### Dingo Optimization Algorithm

The Dingo Optimization Algorithm (DOA) introduces a novel meta-heuristic strategy inspired by the cooperative hunting behavior of dingoes^44^. As a meta-heuristic algorithm, DOA emulates the collaborative hunting approach employed by dingoes in the wild. Its core characteristics involve leveraging dingo-inspired search principles incorporating concepts such as scent marking, exploration, and cooperation into its search strategy^67^. Operating on a population-based approach, the algorithm maintains a collection of solutions, each representing a potential optimization solution. DOA's evolutionary components emulate the cooperative behavior of dingoes through scent marking, serving as an analogy for information sharing among individuals in the population. The algorithm adapts and evolves over iterations, mirroring the dynamic nature of dingo behavior during hunting^67–69^. DOA is an enhancement strategy integrated with the hybrid model's Extreme Learning Machine (ELM). This integration creates a synergistic relationship, introducing adaptability to the learning process of ELM, ultimately contributing to improved convergence and effectiveness in predicting liquefaction resistance.

DOA's principles are more conceptual and inspired by dingo behavior rather than having explicit mathematical equations. The algorithm operates based on the coordination, movement, scent marking, and cooperative strategies observed in dingoes during hunting. While it lacks precise mathematical formulations, DOA utilizes these principles in a population-based approach to iteratively improve solutions to optimize an objective function^70^. Each "iteration" in DOA involves updates to solution positions influenced by concepts derived from dingo behavior, contributing to an evolutionary optimization process. The conceptual representation of the iterative updates in DOA is provided below:

(A) *Iterative update process in DOA (conceptual representation)*
  Movement and coordination: In each iteration, dingoes (representing solutions) coordinate their movements based on scent marking and interactions within the population.
  Solution position: Analogous to the dingo movement, each solution's position is updated within the search space to explore and exploit potential areas that optimize the objective function.
(B) *Scent marking and cooperation*
  Scent marking analogy: Similar to dingo communication through scent marking, solutions share information within the population, influencing each other's movement and decision-making.
  Cooperative strategies: Solutions interact cooperatively by sharing information or influencing each other's positions in the search space, akin to dingoes' cooperative hunting behavior.
(C) *Population-based approach*
  Population initialization: The algorithm starts with an initial population of solutions representing potential solutions to the optimization problem.
  Evolutionary process: Over iterations, solutions evolve and adapt within the search space, driven by the principles inspired by dingo behavior, aiming to optimize the objective function.

In summary, the DOA methodology operates based on principles inspired by dingo behavior, integrating coordination, movement, scent marking, and cooperative strategies observed in dingoes during hunting. Each iteration involves updating solution positions within the search space, influenced by interactions and information sharing akin to dingo behavior^71^. These principles guide the evolutionary optimization process, aiming to iteratively improve solutions toward optimizing the objective function without specific mathematical formulations. The algorithm's effectiveness lies in its conceptual implementation inspired by natural behaviors rather than explicit mathematical equations.

Beyond its application in the hybrid model, DOA exhibits versatility and robustness in tackling a broad spectrum of optimization problems^72^. Drawing inspiration from nature, the algorithm efficiently explores solution spaces, balancing exploration and exploitation. However, DOA's effectiveness may vary depending on the nature of the optimization problem, emphasizing its problem-specific performance. Sensitivity to parameter choices necessitates careful configuration, and the algorithm's relatively recent emergence in the field of meta-heuristics positions it as an area of emerging research with applications across diverse domains^67–69^. In summary, the integration of DOA with ELM showcases an evolutionary approach that enhances the overall capabilities of the liquefaction resistance prediction model.

### Adaptive Neuro-Fuzzy Inference System (ANFIS)

The Adaptive Neuro-Fuzzy Inference System (ANFIS) is a hybrid computational model that combines the adaptability of neural networks with the interpretability of fuzzy logic. It is designed to model complex relationships and patterns within data effectively. ANFIS integrates fuzzy logic systems with neural networks, utilizing a learning algorithm to adjust its parameters and adapt to the underlying data distribution. This adaptability makes ANFIS well-suited for modeling nonlinear systems, pattern recognition, and solving complex decision-making problems. The synergy between fuzzy logic and neural networks in ANFIS provides a robust framework for capturing and interpreting intricate relationships in diverse datasets^73–76^. In ANFIS, membership functions determine how well input data belongs to different fuzzy sets. These memberships are combined to calculate the firing strengths of individual rules, which are then normalized to provide the contribution of each rule to the output, as shown in Eqs. (4–6). The membership grade [*μ*_*i*_ (*x*)] for input data *x* in fuzzy set *i* is calculated using Gaussian or other membership functions, as shown in Eq. (4)^77^. Here, *c*_*i*_ represents the center of the membership function for the fuzzy set *i*; *σ*_*i*_ is the width parameter; *m* is a fuzziness exponent affecting the shape of the membership function.

4 $${\mu }_{i}\left(x\right)=\frac{1}{1+{\left(\frac{x-{c}_{i}}{{\sigma }_{i}}\right)}^{2m}}$$

The firing strength [*w*_*i*_*(x*)] of rule *i* is determined by the product of individual input memberships, as shown in Eq. (5). *n* represents the number of input variables^78^. *μ*_*ij*_ (*x*) denotes the membership grade of input *x* in the *j*th fuzzy set of the *i*th rule.

5 $${w}_{i}\left(x\right)={\prod }_{j=1}^{n}{\mu }_{ij}\left(x\right)$$

The normalized firing strength [*w*_*i*_^∗^(*x*)] is calculated to determine the contribution of rule *i* to the overall output, as shown in Eq. (6). Here, *N* denotes the total number of rules.

6 $${w}_{i}^{*}\left(x\right)=\frac{{w}_{i}\left(x\right)}{\sum_{i=1}^{N}{w}_{i}\left(x\right)}$$

#### ANFIS with fuzzy C-means (ANFIS-FCM)

The ANFIS-FCM hybrid model represents a sophisticated approach to liquefaction resistance prediction in sand-silt mixtures by synergizing the ANFIS with Fuzzy C-Means (FCM) clustering. ANFIS is acknowledged for its adaptability, employing a learning mechanism to adjust fuzzy inference system parameters dynamically^79^. This adaptability is enhanced by the integration of fuzzy logic, providing crucial interpretability that aids in understanding the rationale behind predictions^58,59,80^. On the other hand, FCM contributes a robust clustering strategy, dividing the dataset into fuzzy partitions based on similarity and utilizing membership functions to assign degrees of belongingness to each cluster. This data segmentation capability enhances the model's capacity to capture nuanced relationships within the sand-silt mixture data^81–83^. More specifically, FCM aims to cluster data points by iteratively updating the membership matrix based on the distances between data points and cluster centroids. This process continues until the membership matrix stabilizes, assigning data points to clusters, as shown in Eq. (7)^58,59,80^. Here, *u*_*ij*_ represents the membership of data point *i* in cluster *j*; *c* is the number of clusters; *x*_*i*_ denotes the *i*-th data point; *v*_*j*_ is the centroid of cluster *j*; *m* is a fuzziness parameter (typically set between 1 and 2).

7 $${\mu }_{ij}=\frac{1}{\sum_{k=1}^{c}{\left(\frac{\parallel {x}_{i}-{v}_{j}\parallel }{\parallel {x}_{i}-{v}_{k}\parallel }\right)}^{\frac{2}{m-1}}}$$

Integrating ANFIS and FCM in the hybrid model forms a powerful enhancement strategy. By combining adaptive neuro-fuzzy inference with data-driven clustering, ANFIS-FCM offers a comprehensive modeling ability that captures intricate relationships, providing a nuanced understanding of liquefaction resistance prediction. In the broader context, ANFIS-FCM contributes to the evolutionary approach of the hybrid model, showcasing its role in enhancing adaptability and efficiency in liquefaction resistance prediction^81–83^. The hybrid model, with its advanced optimization and predictive capabilities, offers improved accuracy and insights into the liquefaction resistance of sand-silt mixtures^84^.

#### ANFIS with sub-clustering (ANFIS-sub)

The ANFIS with sub-clustering (ANFIS-sub) hybrid model represents an innovative approach to enhance the accuracy of liquefaction resistance prediction in sand-silt mixtures. Rooted in the ANFIS, the model capitalizes on its renowned adaptability, merging the strengths of fuzzy logic and neural networks to intricately model relationships within the dataset. ANFIS dynamically adjusts its fuzzy inference system parameters through a learning mechanism, fostering adaptability to the underlying patterns in the sand-silt mixture data. Integrating fuzzy logic enhances interpretability, providing valuable insights into the rationale behind predictions^60–62^. ANFIS-Sub introduces a sub-clustering technique to refine the clustering process within the dataset further. This refinement allows for a more granular analysis by breaking down clusters into subgroups, capturing finer distinctions within the sand-silt mixture data. The hybrid model strategically integrates ANFIS with sub-clustering techniques, creating a synergy that leverages adaptive neuro-fuzzy inference and fine-grained sub-clustering. This integration enhances the model's ability to represent complex patterns in liquefaction resistance prediction, contributing to a more accurate and nuanced understanding of the task^85–87^. To emphasize further, Sub-clustering techniques aim to further refine clusters created by the ANFIS membership functions. This refinement involves updating cluster centroids and memberships within clusters to capture finer distinctions within the data. In a Sub-clustering process, the centroids of clusters may be updated iteratively. For example, in the case of K-Means sub-clustering, the centroid *v*_*j*_ of cluster *j* can be updated using Eq. (8)^60–62^. Here, *v*_*j*_ represents the updated centroid of cluster *j*; *N* denotes the total number of data points. *u*_*ij*_ represents the membership of data point *i* in cluster *j*; *x*_*i*_ is the *i*-th data point. The membership grades (*u*_*ij*_) within clusters are recalculated based on the distances between data points and cluster centroids using Eq. (7).

8 $${v}_{j}=\frac{\sum_{i=1}^{N}{\mu }_{ij}\times {x}_{i}}{\sum_{i=1}^{N}{\mu }_{ij}}$$

ANFIS-Sub's accuracy is systematically compared with classic ELM and other ANFIS-based models in the evaluation phase. The comparison serves as a robust benchmark for predictive performance, employing rigorous performance metrics such as accuracy, precision, and recall to comprehensively assess and compare each model's accuracy. In the broader evolutionary approach of the hybrid model, ANFIS-Sub plays a crucial role, contributing to enhanced adaptability and efficiency in liquefaction resistance prediction. In summary, the ANFIS with Sub-clustering (ANFIS-Sub) hybrid model not only advances the accuracy of prediction but also aligns with the overarching evolutionary approach, showcasing its pivotal role in enhancing the scientific rigor of liquefaction resistance prediction methodologies^85–87^.

### Model development

This study investigates the effectiveness of a hybrid model, named ELM-DOA, which combines the ELM algorithm with the DOA algorithm, for predicting the required strain energy to initiate liquefaction in sand-silt mixtures. The DOA algorithm is employed to optimize the parameters of ELM, such as weight and bias values, by minimizing the objective function, which is the root mean square error (RMSE). The DOA algorithm in the ELM-DOA model employs a nature-inspired approach to optimization by mimicking the hunting behavior of dingoes. It iteratively explores the search space by updating the weight and bias values of the ELM model based on fitness evaluation. During each iteration, the algorithm strikes a balance between exploration and exploitation^44^. Exploration allows the algorithm to search a wide range of parameter values, enabling it to discover potentially better solutions in different regions of the search space. Exploitation focuses on refining the parameter values around promising regions that have shown good fitness, aiming to exploit the local information and further improve the model's performance. The DOA algorithm continues this iterative process until a termination criterion is met. The termination criterion could be a predefined number of iterations or a threshold for improvement in fitness. By reaching convergence or meeting the termination criterion, the algorithm ensures that the ELM-DOA model is optimized to the best extent possible within the given search space. By combining exploration and exploitation strategies, the DOA algorithm effectively optimizes the ELM model by finding the optimal weight and bias values that minimize the objective function (RMSE). This optimization process enhances the performance of the ELM-DOA model in predicting the required strain energy for liquefaction in sand-silt mixtures.

**Figure 2:**
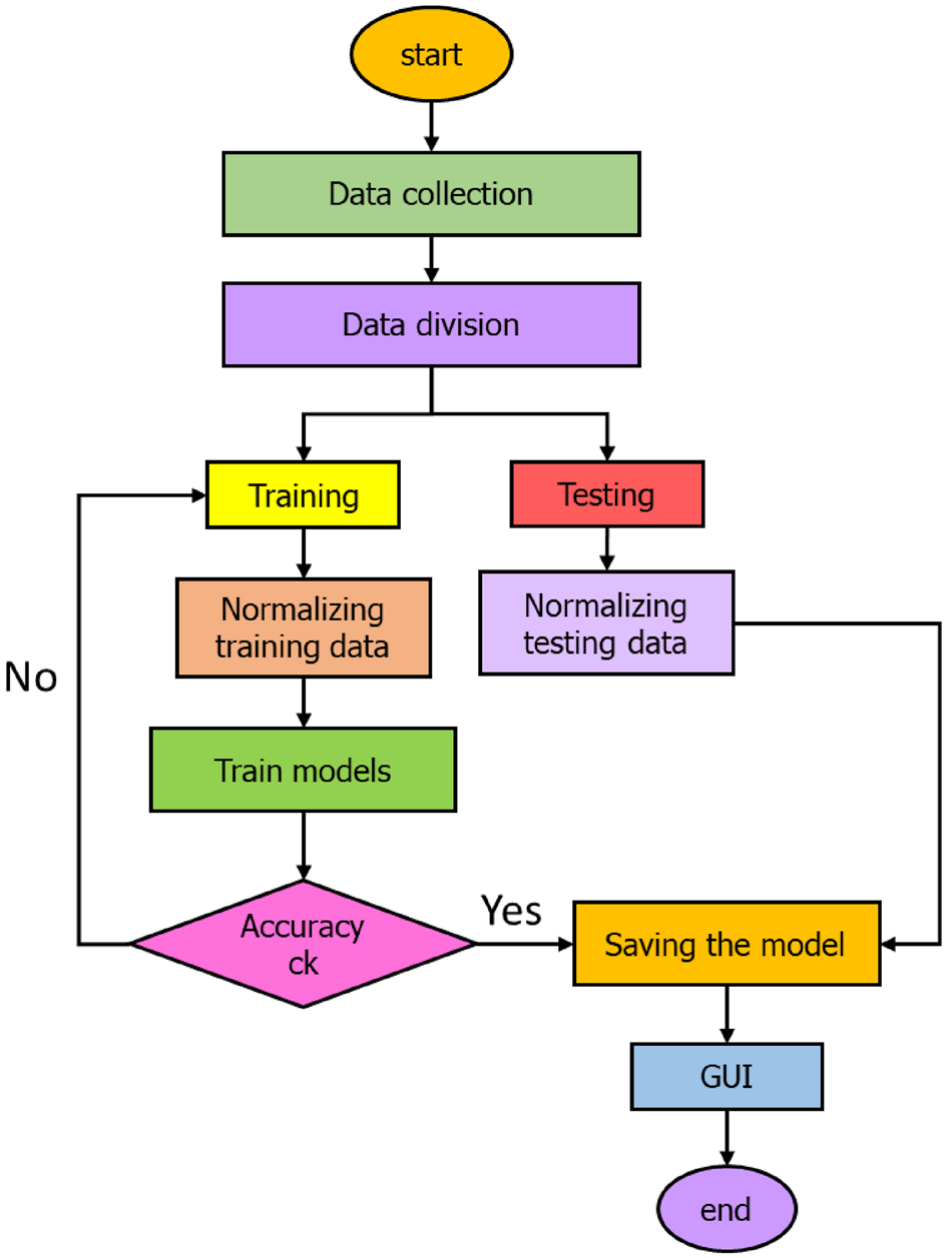
The primary process of model development.

To assess the accuracy and reliability of the hybrid model, it is compared with well-known benchmark models including ANFIS-FCM, classical ELM, and ANFIS-Sub models. The hyperparameters of these models are determined through a trial-and-error approach aimed at minimizing the objective function (*RMSE*). Once the optimal condition is reached, the training process is halted, and the results are saved for future comparison. The development process of the prediction models is illustrated in Fig. 2. It should be noted that the data used in this study is randomly divided into two phases: a training phase, which constitutes 70% of the total collected samples, and a testing phase, which utilizes the remaining 30% of the samples. The applied random division (70/30) is commonly employed in regression-related machine learning studies, as observed in previous works^88–91^. The 70% training data allocation allows for sufficient data to train the model, while the 30% testing data provides ample data for evaluating the model's generalization capabilities. This division strategy achieves a balanced distribution of data between training and testing, facilitating accurate model training and reliable assessment of its performance.

In order to ensure fair feature comparison, two normalization procedures have been employed in this paper. The first procedure involves classic normalization, where features and labels are linearly scaled to fit within the range of zero to one using the following equation (Eq. 9):

9 $${Xn}_{i } = \frac{{X}_{i}-\alpha }{\beta -\alpha }$$

where, ${Xn}_{i}$ is the normalized vector of ith the sample $X$ is the vector that is needed to normalize while, the coefficients $\beta ,$ and $\alpha$ are maximum and minimum values which computed from training data set.

**Figure 3:**
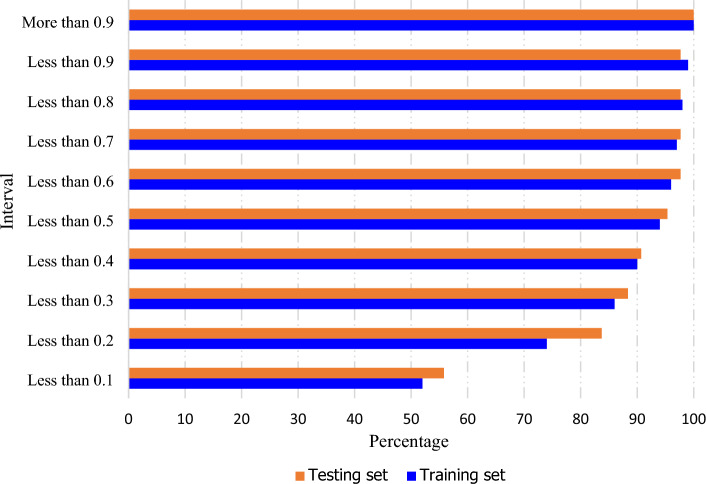
Distribution of hazard and non-hazard events across liquefaction resistance intervals in training and testing sets.

In this study, a random data division procedure was used to split the data into training and testing phases, which is widely employed to minimize bias in previous researches. Avoiding bias between training and testing data is crucial for a machine learning model to possess strong generalization capacity. Comparing the strain energy-based liquefaction resistance between the training and testing data, as shown in Fig. 3, it was observed that the normalized liquefaction resistance values less than 0.1 accounted for approximately 52% in the training set, similar to around 56.8% in the testing set, indicating similar characteristics for lower values in both datasets. Moving to the Less than 0.6 (<0.6) interval, both the training and testing sets covered a higher percentage of the total values, with the training set representing 96% and the testing set representing 97.67% of the total values, indicating a more significant representation of hazard and non-hazard events within this interval. Furthermore, in the More than 0.9 (>0.9) interval, both the training and testing sets encompassed 100% of the total values, providing a complete representation of extreme liquefaction resistance values in both sets. These comparisons highlight the efforts to ensure a fair distribution of hazard and non-hazard events across different intervals as a percentage of the total values in both the training and testing sets. Consequently, the applied approach effectively mitigates zero sampling bias and enhances the reliability of the model's predictions.

The second scenario involves non-linear normalization, which includes applying a logarithm base 10 to the data before using the aforementioned equation. Subsequently, the data is linearly scaled between zero and one using the same formula. Then, a de-normalizing procedure is proposed to return data to its normal scales. Then, a de-normalization procedure is employed to restore the data to its original scales^92^. Lastly, (GUI) has been developed, specifically tailored to the best prediction model, to aid engineers and researchers in effectively utilizing the best predictive model of this study.

### Statistical metrics

Seven statistical performance indices have been applied in this study to assess the accuracy of the prediction models. These indices serve as metrics to quantify the prediction errors and assess the match between predicted values and measured values^93^. The metrics used include (*RMSE*), mean absolute error (*MAE*), maximum absolute percentage relative error (erMax), mean absolute percentage error (*MAPE*), and uncertainty at 95% (*U*_*95*_). Additionally, other metrics such as the correlation coefficient (*R*), Willmot index (*WI*), and Nash coefficient (*NSE*) have been utilized to gauge the accuracy of the predictions and determine how well they align with the measured values. The mathematical expressions of the applied metrics are provided below (Eqs. 10–17)^94–96^:

10 $$MAPE\% = \frac{100}{n}\sum_{i=1}^{n}\left|\frac{{X}_{{obs}_{i}}-{X}_{{pred}_{i}}}{{X}_{{obs}_{i}}}\right|$$

11 $$RMSE= \sqrt{\frac{1}{n}\sum_{{\text{i}}=1}^{n}{\left({X}_{{obs}_{i}}-{X}_{{pred}_{i}}\right)}^{2}}$$

12 $$MAE= \frac{1}{n}\sum_{i=1}^{n}\left|{X}_{{obs}_{i}}-{X}_{{pred}_{i}}\right|$$

13 $$R=\frac{\sum_{{\text{i}}=1}^{{\text{n}}}\left[\left({X}_{{obs}_{i}}-\overline{{X }_{obs}}\right)\left({X}_{{pred}_{i}}-\overline{{X }_{pred}}\right)\right]}{\sqrt{\sum_{{\text{i}}=1}^{{\text{n}}}{\left({X}_{{obs}_{i}}-\overline{{X }_{obs}}\right)}^{2}\sum_{{\text{i}}=1}^{{\text{n}}}{\left({X}_{{{\text{pred}}}_{{\text{i}}}}-\overline{{X }_{pred}}\right)}^{2} }}$$

14 $$WI =1- \frac{\sum_{{\text{i}}=1}^{{\text{n}}}({X}_{{obs}_{i}}-{X}_{{pred}_{i}}{)}^{2}}{\sum_{{\text{i}}=1}^{{\text{n}}}(\left|{X}_{{pred}_{i}}- \overline{{X }_{obs}}\right|{+ \left|{X}_{{obs}_{i}}- \overline{{X }_{obs}}\right|)}^{2}}$$

15 $$NSE=1-\frac{\sum_{i=1}^{n}\left|{X}_{{obs}_{i}}-{X}_{{pred}_{i}}\right| }{\sum_{i=1}^{n}\left|{X}_{{obs}_{i}}-\overline{{X }_{obs}}\right|}$$

16 $$erMax = max \left(\left|\frac{{X}_{{obs}_{i}}-{X}_{{pred}_{i}}}{{X}_{{obs}_{i}}}\right|\right)$$

17 $${U}_{95} = 1.96 {\left({RMSE}^{2} + {SD}^{2}\right)}^{0.5}$$

where, the *n,* ${X}_{{obs}_{i}}-{X}_{{pred}_{i}}$, are representing the total number of samples, measured value of i th sample, and predicted value, respectively. Also, the terms $\overline{{X }_{obs}}$, and $\overline{{X }_{pred}}$ are the mean values of observed and predicted values. Finally, the *SD* is the standard deviation of the forecasted errors.

## Modeling result

### Prediction results: both scenarios

**Figure 4:**
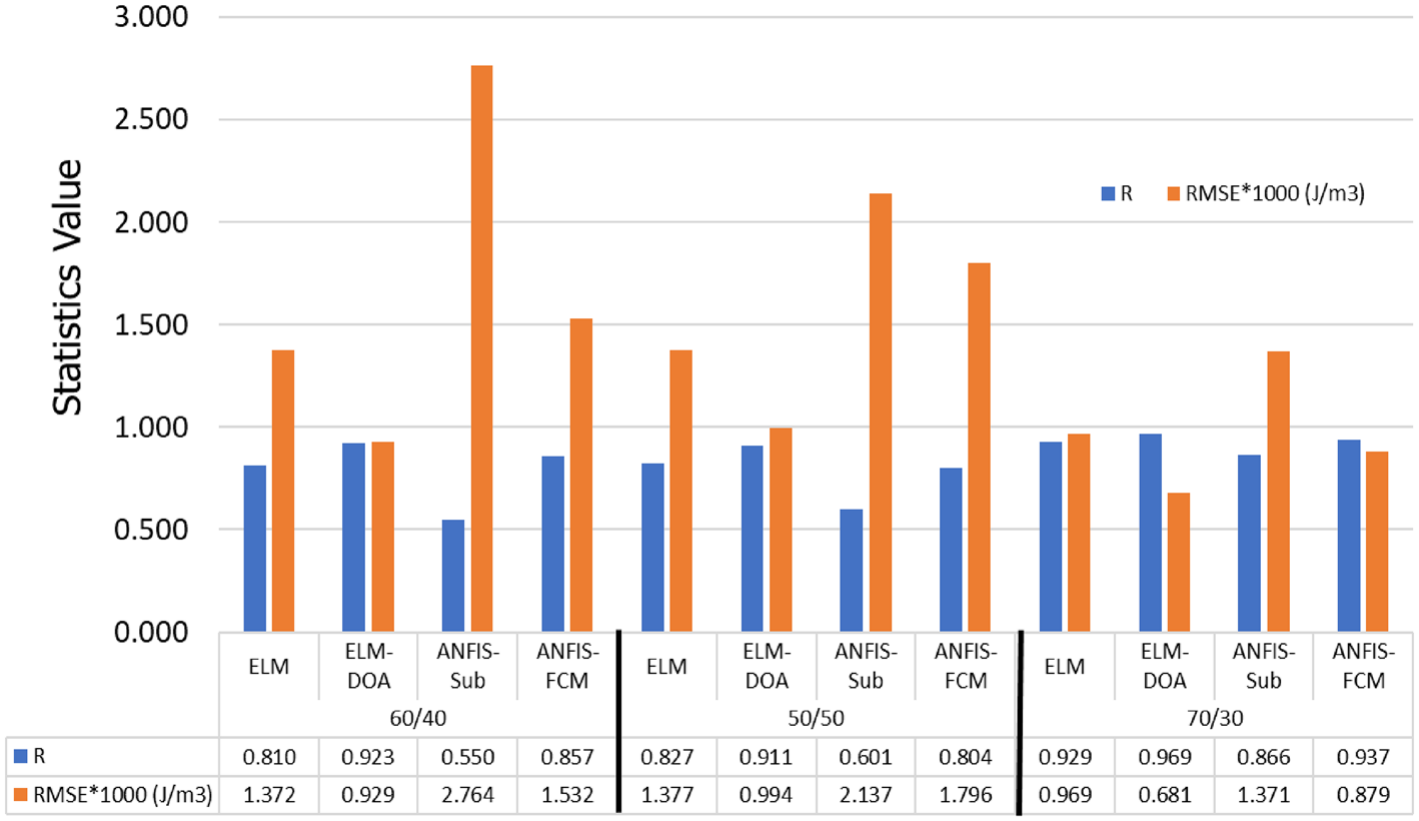
Comparing model performance with varying training/testing ratios for liquefaction resistance prediction.

The estimation of liquefaction resistance, a key parameter indicating soil’s capacity to withstand temporary liquid-like behavior during cyclic loading, was performed using four distinct machine learning techniques: ELM, ELM-DOA, ANFIS-Sub, and ANFIS-FCM. In order to maximize the effectiveness of our modeling process, the performances of our models were rigorously evaluated across distinct phases, encompassing both training and testing stages, under two varied scenarios of data preprocessing (Tables 2 and 3). Furthermore, it is important to note that the hyperparameters for all models are provided in Table S-3 in the Supplementary File. Notably, in the first scenario, linear data normalization was employed, whereas in the second scenario, non-linear data normalization was utilized. The utilization of metrics such as *MAE, RMSE, MAPE, R, erMax, NSE,* and *WI* facilitate a thorough examination of the model's effectiveness. It is noteworthy that higher *R, NSE*, and *WI* values signify a stronger correlation between the predicted and actual values, indicating the model's robust performance. Additionally, lower *RMSE*, *MAE, MAPE, and erMax* values further reinforce the accuracy of the model, as they indicate minimal discrepancies between predicted and observed values. The results obtained from both scenarios reveal that the ANFIS-based models suffer from overfitting. This is evident in the lower prediction accuracy during the testing phase and higher accuracy during the training phase, as compared to other models. Also, the prediction accuracy of the models significantly improved when using nonlinear normalization (scenario 2) compared to classical linear normalization, as stated in scenario one. The findings presented in Table 2 reveal that all the ML models showed good predictive performance, particularly the ELM-DOA model, which exhibited slightly better results compared to all other models under scenario-1 during the training and testing stages with *MAE* = 623.713 J/m^3^ and 505.821, *RMSE* = 952.048 J/m^3^ and 680.956 J/m^3^, *MAPE* = 30.750 and 39.950, *R* = 0.896 and 0.969, *erMax* = 1.291 and 2.251, *NSE* = 0.582 and 0.665 and *WI* = 0.943 and 0.982, respectively. For further assessment, additional analyses were conducted using different proportions of the training set and testing set, including a 50:50 split and a 60:30 split, in addition to the standard 70:30 ratio. The quantitative results are summarized in Fig. 4. The findings consistently indicate that the 70:30 training/testing ratio yielded the best outcomes, leading to improved performance across all models. This ratio demonstrated good accuracy in predicting liquefaction resistance. In contrast, the other ratios resulted in less accurate predictions. These findings suggest that a higher proportion of training data (70%) in comparison to testing data (30%) is preferable for achieving optimal performance in the prediction of liquefaction resistance.

**Table 2:** The performance of the proposed models during the training phase: Classic normalization (scenario-1).

Data mode	Models	Statistical parameters						
		MAE (J/m^3^)	RMSE (J/m^3^)	MAPE%	R	erMax	NSE	WI
Training	ELM-DOA	623.713	952.048	30.75	0.896	1.291	0.582	0.943
	ELM	800.894	1182.843	42.00	0.834	2.202	0.463	0.902
	ANFIS-Sub	9.668	55.580	0.410	1.000	0.129	0.994	1.000
	ANFIS-FCM	464.033	737.395	22.76	0.939	0.884	0.689	0.968
Testing	ELM-DOA	505.821	680.956	39.950	0.969	2.251	0.665	0.982
	Classic ELM	730.990	969.364	56.770	0.929	2.489	0.516	0.957
	ANFIS-Sub	757.163	1371.102	43.920	0.866	1.964	0.499	0.886
	ANFIS-FCM	574.232	879.408	39.880	0.937	2.289	0.620	0.966

**Table 3:** The performance of the proposed models during the training phase: Log normalization (scenario-2).

Data mode	Models	Statistical parameters						
		MAE (J/m^3^)	RMSE (J/m^3^)	MAPE%	R	erMax	NSE	WI
Training	ELM-DOA	468.965	834.157	17.600	0.923	0.961	0.686	0.956
	ELM	475.853	846.841	17.600	0.920	1.015	0.681	0.954
	ANFIS-Sub	158.861	316.836	5.700	0.989	0.681	0.894	0.994
	ANFIS-FCM	353.617	687.021	14.200	0.949	0.591	0.763	0.971
Testing	ELM-DOA	335.162	484.286	24.900	0.983	1.021	0.778	0.991
	Classic ELM	404.416	556.129	28.200	0.979	1.100	0.732	0.988
	ANFIS-Sub	679.824	1014.447	51.500	0.916	3.079	0.550	0.954
	ANFIS-FCM	512.849	857.677	27.700	0.958	1.072	0.660	0.963

In scenario-2, the ELM-DOA model demonstrated superior performance with higher *NSE* (0.686 and 0.778), *R* (0.923 and 0.983), and *WI* (0.956 and 0.991), and lower *MAE* (468.965 J/m^3^ and 335.162 J/m^3^), *RMSE* (834.157 J/m^3^ and 484.286 J/m^3^), *MAPE* (0.176 and 0.249) and *erMax* (0.961 and 1.021) for both training and testing phases (Table 3). The reported results highlight the remarkable accuracy achieved by the proposed models in both the training and testing phases. This serves as strong evidence supporting the validity of the optimization process employed. During the learning phase, the models effectively minimize the objective function (*RMSE*), indicating their ability to capture the underlying patterns and relationships within the data. Consequently, during the testing phase, the models showcase a high level of accuracy in their predictions, further affirming their reliability and effectiveness.

**Figure 5:**
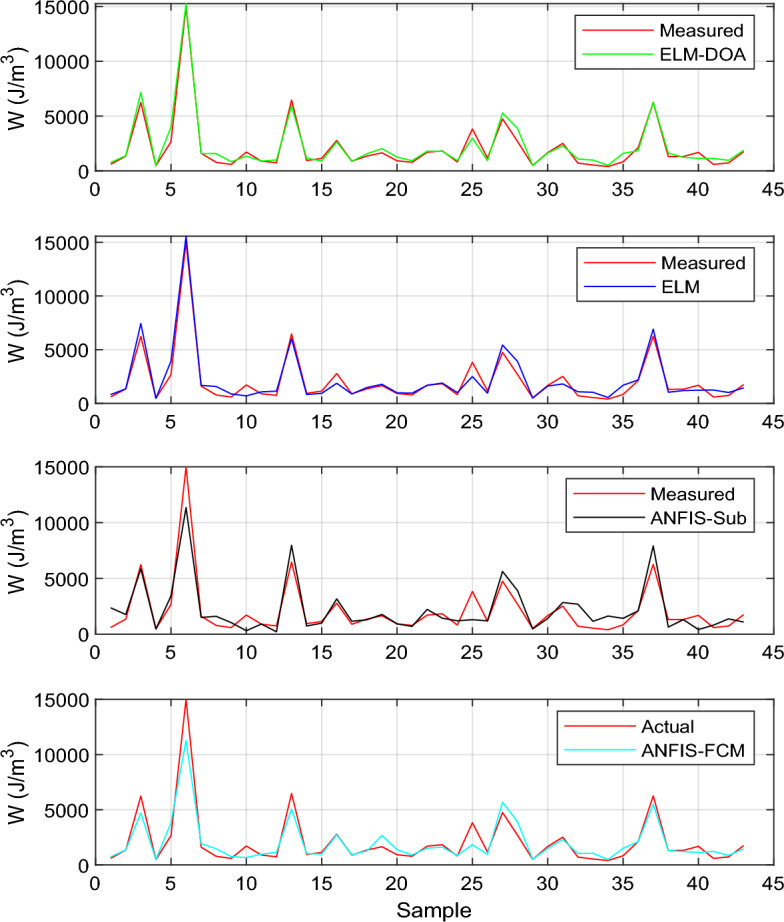
Variation of Predicted values of liquefaction resistance using ELM-DOA, ELM, ANFIS-sub, and ANFIS-FCM in comparison to actual liquefaction resistance values.

Figure 5 displays the performance plot of all the models during the testing phase for the second scenario. It is observed from the figure that the prediction pattern of all the models exhibits commendable conformity with the actual data pattern. Notably, the ELM-DOA model's predictions display superior agreement and align more closely with the observed patterns compared to the other models. This suggests that the ELM-DOA model performs exceptionally well in capturing and predicting the underlying patterns during the testing phase, highlighting its efficacy in this context. The graph underscores the reliability of this hybrid ELM-DOA model in accurately reflecting the observed data trends.

**Figure 6:**
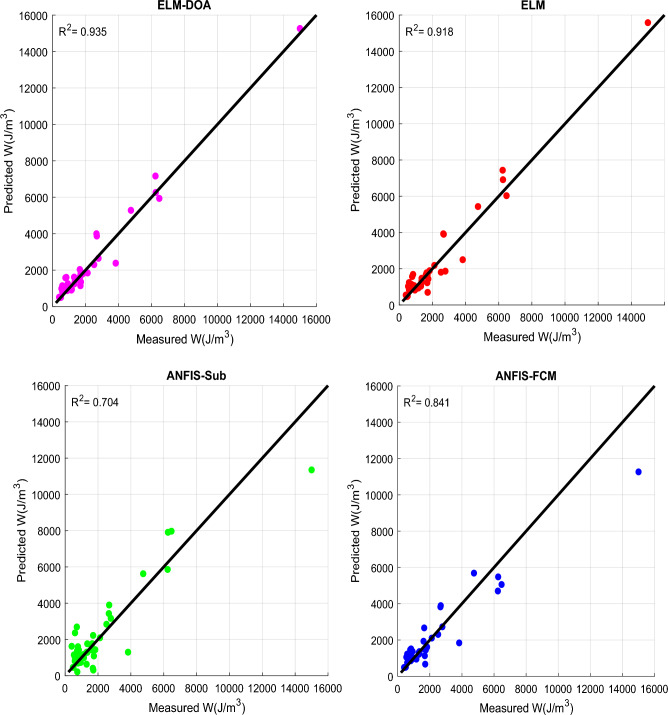
Scatter plots of observed and predicted liquefaction resistance values for all the considered models for testing dataset.

The scatter details of the observed and the predicted liquefaction resistance values for all the developed models during the testing phase are shown in Fig. 6. Notably, all models exhibit a good fit with the observed data. It is worth highlighting that the ELM-DOA model, characterized by the highest *R*^*2*^ value (0.935), displays a particularly denser scattering pattern around the isoline regression line in comparison with the other ML models. This phenomenon underscores the robust performance of the ELM-DOA model, as the tight clustering signifies a high level of accuracy and reliability in predicting liquefaction resistance values.

**Figure 7:**
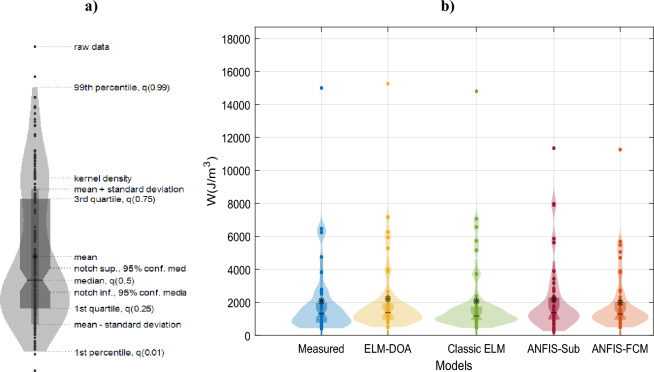
Violin cum box plot for applied ML techniques. (**a**) a visual representation highlighting the essential characteristics of the violin plot^97^, and (**b**) a comparison between the observed values and the corresponding predictions generated by ML models.

The Violin cum box plot is a unique graphical representation that seamlessly merges the violin plot and box plot, featuring the advantages of both. This hybrid plot displays multiple layers and includes a mean marker, akin to the traditional violin and box plots (see Fig. 7a)^92^. Moreover, Fig. 7b displays the violin and box plots for various ML models (ELM-DOA, ELM, ANFIS-sub and ANFIS-FCM) used to estimate liquefaction resistance values for the testing datasets. It was observed that the violin representing the observed values closely mirrors that of the ELM-DOA-based violin in comparison to other models for the testing data. The ELM-DOA box exhibits trends identical to those of the observed value’s box, characterized by an equivalent mean value (represented by an asterisk in Fig. 7). Furthermore, the outer layer of the violin cum box plot for both the actual and ELM-DOA model demonstrates a notable degree of symmetry when compared to other machine learning models in estimating liquefaction resistance values. It is important to note that, the ELM-DOA model demonstrates more efficient prediction of extreme values compared to other models. It can be concluded that the ELM-DOA model performs the best, followed by the ELM model, while the other two ANFIS models exhibit poor performance.

**Figure 8:**
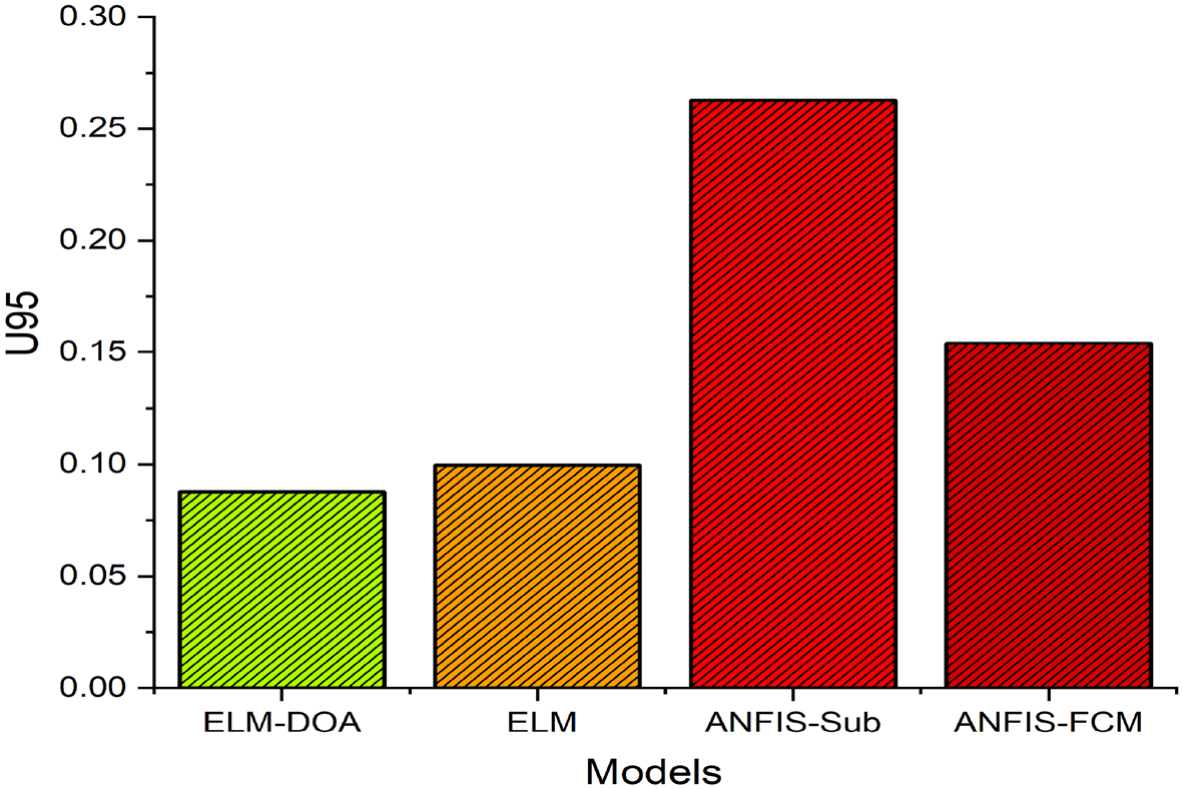
U_95_ values for the applied ML models.

The uncertainty analysis is performed to assess the level of confidence in a model's predictions. This analysis helps in assessing the reliability and robustness of the proposed models by quantifying the potential range of true values for the predicted outcomes. The *U*_*95*_ interval represents the range where it is expected to find the true value for approximately 95% of similar experiments. Figure 8 demonstrates the results of the uncertainty analysis attained for the applied models i.e., ELM, ELM-DOA, ANFIS-sub and ANFIS-FCM. The results presented in the bar chart (Fig. 8) reveal that ELM-DOA demonstrates the lowest uncertainty, boasting a normalized U95 value of 0.0878, outperforming ELM (*U*_*95*_ = 0.099), ANFIS-sub (*U*_*95*_ = 0.262) and ANFIS-FCM (*U*_*95*_ = 0.154). These findings underscore the superior stability and consistency of ELM-DOA, indicating its reduced sensitivity compared to other machine learning models concerning input data variations.

**Figure 9:**
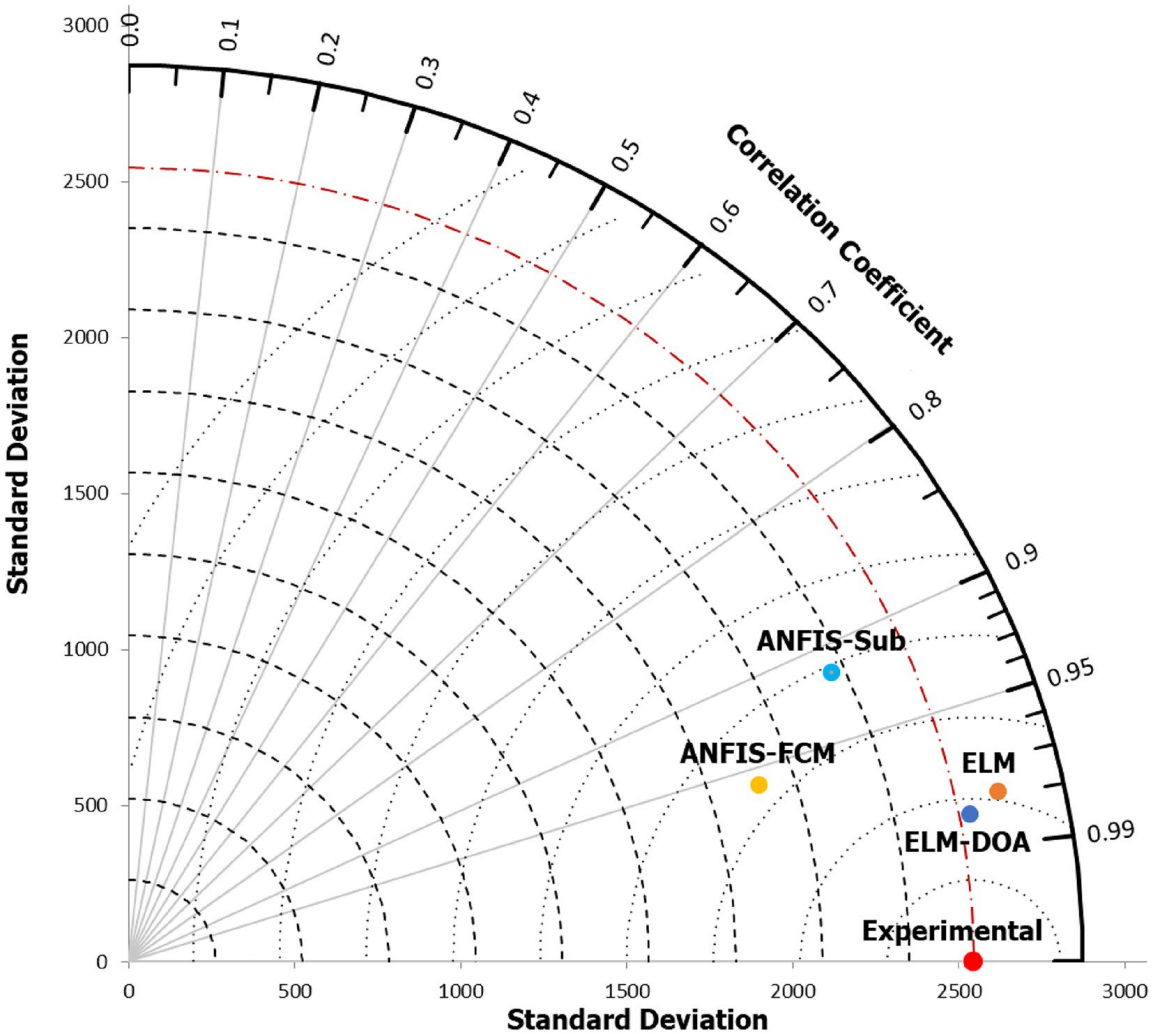
Comparative analysis of prediction models and measured liquefaction resistance using a Taylor Diagram.

The Taylor diagram visually compares model performances using three key statistical metrics—correlation coefficient (*R*), standard deviation (*SD*), and root-mean-square error (*RMSE*)—in a 2-D plot, assessing their simultaneous variations and highlighting distance-based distinctions between the observed and predicted data. The depicted liquefaction resistance data is represented as a point on the x-axis with *R* = 1 and *SD* = 2500 (Fig. 6). Based on Fig. 9, it is evident that all four models demonstrated strong prediction performance during the testing stage, exhibiting a correlation coefficient exceeding 0.90 and SD under 2700 (with all model predictions falling within the SD curve). Notably, the ELM-DOA model (represented by the dark blue point), positioned closest to the observed data point, exhibited exceptional accuracy and outperformed other ML models in predicting liquefaction resistance values. Overall, the developed models exhibited exceptional performance throughout their training and testing phases, consistently surpassing benchmarks across various statistical metrics and graphical analyses. Their ability to accurately predict outcomes aligned closely with actual values, underscoring their robust generalization capacity. Notably, these models showcased a remarkable consistency in their predictions, illustrating their reliability across diverse datasets.

### Validation of ELM-DOA with previous works

Hybrid ML models have been widely employed to estimate the probability of liquefaction on a regional or global scale. However, this study breaks new ground by employing a novel predictive ensemble model to enhance liquefaction potential in sand-silt mixtures. The proposed ELM-DOA method yielded enhanced liquefaction resistance estimates compared to other methods in the literature. Its results demonstrated statistical performance on par with or superior to alternative approaches, highlighting its effectiveness in accurately predicting liquefaction resistance values. For example, some researchers developed numerous hybrid ML models for the prediction of liquefaction probability in soils^98^. The performance, accuracy, and reliability of the models were evaluated using different assessment and evaluation tools. The hybrid model with an *R*^*2*^ = 0.713 presented the highest accuracy in terms of prediction of  the liquefaction probability in soils. Moreover, other investigation reported the development of two hybrid models based on Support Vector Machines, Radial Basis Function and Grey wolf optimization algorithm^32^. The models were developed on the inputs of earthquake magnitude, water table, total vertical stress and other important parameters. The results of the study suggested that the hybrid models with higher R^2^ values (0.757 and 0.692) outperformed the other models in terms of prediction accuracy. Furthermore, other study applied advanced hybrid ML models for forecasting soil liquefaction potential for railway embankments built on fine soil deposits^99^. The moisture content, wet density, dry density, liquid limit, plastic limit, and plasticity index were incorporated as inputs for the model development. Later it was concluded that the developed hybrid model (ANFIS-FF) showed good prediction accuracy in terms of *R*^*2*^ (0.900) for liquefaction prediction. Overall, the proposed hybrid model (ELM-DOA) in this study demonstrates higher accuracy in predicting liquefaction resistance, with an *R*^*2*^ value exceeding 0.93. This indicates the superiority of the models developed in previous work.

### Graphical User Interface (GUI)

**Figure 10:**
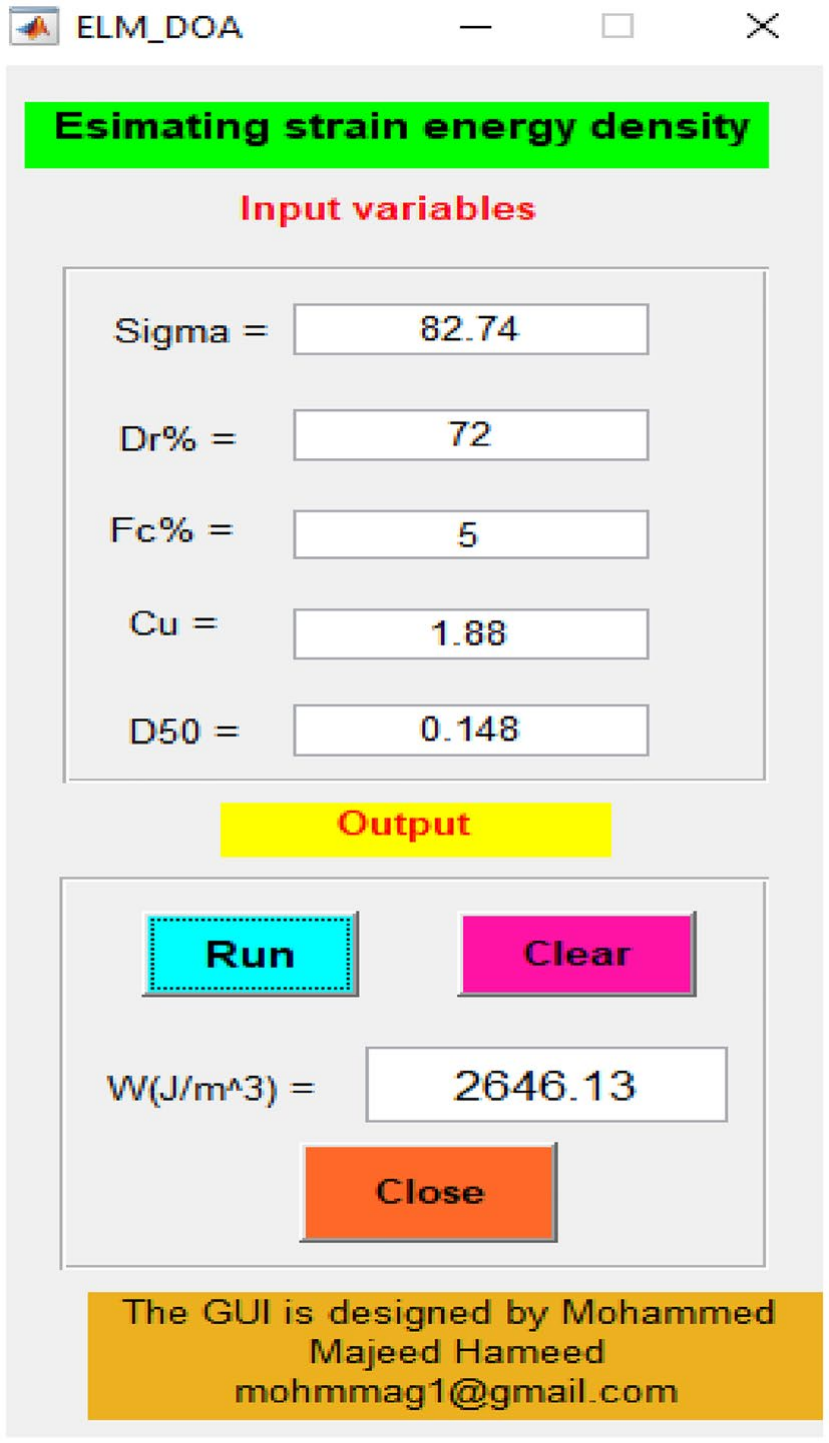
GUI of the ELM-DOA model based on liquefaction resistance value prediction.

As mentioned in the preceding section, the suggested ELM-DOA model exhibited significant promise in estimating the strain energy-based liquefaction resistance. Given the nature of hybrid machine learning models, the integration into daily engineering practice necessitates a bridge between complex algorithms and user-friendly applications. To fulfill this requirement, we developed a MATLAB-based graphical user interface (GUI) for the ELM-DOA model, which is demonstrated in Fig. 10. This GUI is not just a testament to the model's expansive practical application but also a crucial tool to facilitate its use among engineers who may not have specialized expertise in hybrid ML models. Figure 7 shows a graphical illustration that delineates the extensive scope of applicability for the proposed ELM-DOA model. Through the effective utilization of the GUI software, we successfully conducted precise predictions for liquefaction resistance (W = 2646.13) at sigma = 82.74, Dr% = 72, Fc% = 5, Cu = 1.88 and D50 = 0.148. The designed feature substantially augments the practical and academic value of the research, as it provides a standardized platform for performance assessment and comparison, streamlining future advances in the field.

## Discussion

The study revealed that integrating DOA and ELM in a hybrid model yielded markedly superior performance in predicting liquefaction resistance compared to individual ML models such as ELM. The ELM-DOA model serves to augment the predictive precision of the conventional ELM by meticulously ascertaining the most advantageous weights and bias values. Through comprehensive evaluation based on statistical criteria and graphical assessments, the hybrid model (ELM-DOA) not only surpasses the performance of the traditional ELM but also outshines other widely recognized hybrid models, including ANFIS-sub, and ANFIS-FCM. This aligns with similar findings in scientific literature, highlighting the efficacy of hybrid models in enhancing predictive accuracy for simulating liquefaction resistance values^100^. The findings presented in the study contribute to the growing body of evidence supporting the advantages of hybrid models in addressing complex geotechnical engineering challenges such as stress–strain modeling of soils, analysis of piles bearing capacity, assessment of settlement in shallow foundations, investigation into earthquake-induced liquefaction, and scrutiny of seismic lateral spreading phenomena^33,101^.

The Dingo Optimization Algorithm (DOA), a novel bio-inspired approach, addresses optimization problems by emulating the social behavior of Australian dingoes. Drawing inspiration from dingo hunting techniques involving persecution, grouping, and scavenging, DOA enhances overall efficiency through three search strategies and four formulated rules. This combination achieves a delicate equilibrium between exploitation and exploration across the search space. The proposed model outperforms other individual models developed in the present study for predicting liquefaction resistance values in a sand-silt mixture, effectively. Its unique blend of dingo-inspired strategies positions it as a promising solution for such prediction challenges, showcasing its superiority in comparison to other comparable models.

Accurate prediction of soil liquefaction induced by seismic loads is crucial for geotechnical engineers due to its potential for uncontrolled lateral spreading, posing significant threats to regional civil engineering projects. Incidents of liquefaction can compromise the stability of structures, heightening the risk to infrastructure in the area. While conventional in situ tests like the Standard Penetration Test and Cone Penetration Test have been conducted, their susceptibility to uncertainties has prompted a shift towards more accurate methodologies. Consequently, geotechnical engineers are increasingly turning to advanced techniques, including machine learning (ML). ML's precision in analyzing complex data enables a more nuanced evaluation of soil behavior during seismic events, offering a refined understanding of liquefaction risks. This transition to ML applications underscores a progressive approach, empowering engineers to enhance assessments, optimize designs, and implement targeted mitigation strategies in seismic-prone regions. The hybrid ML-model (ELM-DOA) developed in our study has shown excellent performance, as evidenced by higher values *NSE* (0.778), *R* (0.983) and *WI* (0.991), and lower values of *MAE* (335.162), *RMSE* (484.286), *MAPE* (0.249) and *erMax* (1.021).

The results indicate that only the ELM-DOA and ELM models demonstrate good generalization capacity, whereas the ANFIS models encounter difficulties in producing accurate estimates during the testing phase. Additionally, the study findings reveal that the generalization capacity of all models is significantly enhanced when non-linear normalization is applied. This is attributed to the fact that non-linear normalization reduces the data variance, thereby facilitating effective model training and generating more accurate estimates. To illustrate this point, considering the ELM-DOA model as the best one, the *MAE* during the testing phase using linear normalization was 505.821 J/m^3^. However, after applying nonlinear normalization, the *MAE* improved to 335.162 J/m^3^, indicating an increase in prediction accuracy of approximately 33.74%. The other ML models achieved varied improvements, with the ELM model experiencing a 44.68% improvement as the *MAE* reduced from 730.990 to 404.416 J/m^3^, while the ANFIS-Sub model showed a 10.21% improvement, and the ANFIS-FCM model demonstrated a 10.69% improvement. Overall, applying nonlinear normalization improved the overall accuracy of all models by approximately 25%. This improvement was observed across all the applied models, indicating the effectiveness of nonlinear normalization in enhancing the prediction performance of these models. In addition to evaluating our models against internal benchmarks, a comparative analysis with respect to previous iterations developed in earlier studies was conducted. This comprehensive examination offered valuable insights into the evolution and refinement of the modeling techniques, ultimately affirming the success of the applied optimization process.

The results highlight that the ELM-DOA model represents a robust, accurate, and intelligent methodology for predicting soil liquefaction. The model showcases its effectiveness not only in geotechnical engineering but also holds the potential to serve as a valuable tool in various other engineering disciplines. The integration of advanced optimization techniques, such as Dingo Optimizer, with ELM, enhances the predictive capabilities, making it reliable in capturing the intricate patterns associated with soil liquefaction dynamics.

## Conclusion

In this study, the predictive capabilities of the suggested hybrid model (ELM-DOA) were evaluated for predicting strain energy requirements necessary to initiate liquefaction in sand-silt mixtures. To comprehensively evaluate its effectiveness, the performance of ELM-DOA was compared with the standalone ELM model and adaptive neuro-fuzzy inference with fuzzy c-means (ANFIS-FCM), and sub-clustering (ANFIS-Sub) models, respectively. Also, two data preprocessing scenarios are utilized in this work. The first scenario is traditional linear normalization, and the second scenario is non-linear normalization. The findings indicate that non-linear normalization significantly improves the prediction performance of all models by approximately 25% compared to linear normalization. The results demonstrated that the ELM-DOA model outperformed the other models in accurately predicting the liquefaction resistance values. Moreover, the ELM-DOA model, created by integrating the novel metaheuristic algorithm (DO) with ELM, exhibited superior performance compared to standalone ELM, and other models. It achieved a remarkable minimum forecasting error of *RMSE* = 505.821 and 335.162 during both scenarios 1 and 2, respectively. The ELM-DOA model, developed using a novel metaheuristic algorithm (DO) and ELM, performed significantly better than the standalone ELM model and other hybrid models. The ELM-DOA model depicting minimum forecasting errors of *RMSE* = 505.821 and 335.162, was 30% and 17% more accurate than the standalone ELM model during scenarios 1 and 2, respectively. Similarly, the ELM-DOA model showcased improvements of 33% and 50.60% in forecasting accuracies during scenarios 1 and 2 compared to the ANFIS-Sub model. Finally, in the case of ANFIS-FCM, the ELM-DOA presented an improved accuracy of 11.91% and 34.64% during both scenarios. Overall, this study suggests that the ELM-DOA hybrid model is a promising approach for accurately predicting liquefaction values, which is crucial for making informed decision-making in geotechnical engineering.   Also, it may facilitate the development of resilient and sustainable solutions in the face of potential challenges posed by seismic activities and other geological factors.

## Limitations and recommendations

Soil liquefaction prediction research encounters several limitations. Firstly, the reliance on limited data for training and testing models may not fully capture the complexities and variability of real-world scenarios. However, acquiring high-quality, comprehensive real-life datasets is challenging, as it requires extensive fieldwork, laboratory testing, and data collection. This limitation restricts the availability of diverse and representative datasets for model development and validation. Lastly, complex hybrid models, such as ELM-DOA, face challenges in terms of interpretability, making it difficult to understand the underlying mechanisms driving their predictions. Thus, Enhancing the model's interpretability is crucial for establishing trust and confidence among stakeholders, including geotechnical engineers, researchers, and decision-makers. consequently, the recommendations of this study are:

1. Foster collaborative data sharing initiatives within the geotechnical engineering community to facilitate the creation of comprehensive and diverse datasets for model development and validation.
2. Explore interpretable ML techniques or model-agnostic interpretability methods to shed light on the decision-making process of complex hybrid models.
3. Conduct further studies to evaluate the robustness and generalization capabilities of ML models across different geological and environmental conditions.
4. Continuously refine and improve optimization algorithms, such as the Dingo Optimization Algorithm, to enhance the performance and efficiency of hybrid ML models for soil liquefaction prediction.

### Supplementary Information

Supplementary Tables.

## Data Availability

The datasets used and/or analysed during the current study available from the corresponding author on reasonable request. Additionally, all the experimental samples utilized in this study can be found in the Supplementary File, namely Tables S-2 and S-3.
