# Supplementary material for: Investigating a hybrid extreme learning machine coupled with Dingo Optimization Algorithm for modeling liquefaction triggering in sand-silt mixtures
Source: Sci Rep. 2024 May 11;14:10799. doi: 10.1038/s41598-024-61059-6 (PMC11088631; doi:10.1038/s41598-024-61059-6)
Supplement: Supplementary file 1 — Supplementary Tables. [file 41598_2024_61059_MOESM1_ESM.docx]

Table S-1- Training data set

| Sigma c | Dr(%) | FC (%) | Cu | D50(mm) | W (J/m3) |
| --- | --- | --- | --- | --- | --- |
| 80 | 45.9 | 0 | 2.27 | 0.15 | 1286.4 |
| 100 | 23.44 | 0 | 1.52 | 0.23 | 780 |
| 28.8 | 51 | 0 | 1.67 | 0.26 | 550 |
| 16.1 | 67.2 | 28 | 5.88 | 0.13 | 401 |
| 100 | 56.83 | 15 | 12.78 | 0.215 | 1110 |
| 100 | 57.81 | 0 | 1.52 | 0.23 | 3010 |
| 100 | 35.94 | 0 | 1.52 | 0.23 | 1570 |
| 29.3 | 78.7 | 0 | 1.67 | 0.26 | 1160 |
| 100 | 71.14 | 25 | 28.12 | 0.2 | 1880 |
| 100 | 52.8 | 15 | 12.78 | 0.215 | 2030 |
| 82.74 | 71 | 45 | 9.19 | 0.0865 | 2542 |
| 124.11 | 62 | 5 | 1.88 | 0.148 | 3045 |
| 100 | 67.7 | 25 | 28.12 | 0.2 | 2410 |
| 100 | 82.03 | 25 | 28.12 | 0.2 | 3010 |
| 15.24 | 72 | 28 | 5.88 | 0.13 | 414 |
| 33.6 | 51 | 0 | 1.67 | 0.26 | 710 |
| 80 | 62.7 | 0 | 2.27 | 0.15 | 1355.9 |
| 400 | 60.42 | 0 | 1.52 | 0.23 | 8110 |
| 82.74 | 70 | 15 | 2.77 | 0.135 | 1383 |
| 82.74 | 68 | 35 | 4.33 | 0.107 | 1134 |
| 400 | 67.7 | 25 | 28.12 | 0.2 | 4090 |
| 100 | 24.48 | 0 | 1.52 | 0.23 | 1030 |
| 41.4 | 60 | 45 | 9.19 | 0.0865 | 1223 |
| 100 | 83.12 | 25 | 28.12 | 0.2 | 3130 |
| 160 | 42.3 | 0 | 2.27 | 0.15 | 3385.4 |
| 100 | 41.3 | 15 | 12.78 | 0.215 | 960 |
| 34.4 | 66.5 | 0 | 2.27 | 0.15 | 935 |
| 31.3 | 62.8 | 28 | 5.88 | 0.13 | 460 |
| 41.4 | 68 | 15 | 2.77 | 0.135 | 1210 |
| 132.7 | 45.9 | 0 | 2.27 | 0.15 | 1316 |
| 100 | 48.44 | 0 | 1.52 | 0.23 | 2830 |
| 100 | 80.73 | 0 | 1.52 | 0.23 | 5030 |
| 124.11 | 77 | 0 | 1.88 | 0.148 | 7130 |
| 100 | 91.78 | 60 | 25.93 | 0.029 | 5740 |
| 100 | 34.16 | 15 | 12.78 | 0.215 | 660 |
| 100 | 64.58 | 0 | 1.52 | 0.23 | 2140 |
| 100 | 86.44 | 60 | 25.93 | 0.029 | 3160 |
| 82.74 | 67 | 15 | 2.77 | 0.135 | 1380 |
| 100 | 65.1 | 0 | 1.52 | 0.23 | 2120 |
| 100 | 68.23 | 0 | 1.52 | 0.23 | 3510 |
| 100 | 59.38 | 0 | 1.52 | 0.23 | 3750 |
| 124.11 | 74 | 35 | 4.33 | 0.107 | 1645 |
| 160 | 61.4 | 0 | 2.27 | 0.15 | 9023.5 |
| 400 | 40.63 | 0 | 1.52 | 0.23 | 4220 |
| 160 | 42.6 | 0 | 2.27 | 0.15 | 1788.7 |
| 100 | 28.57 | 15 | 12.78 | 0.215 | 590 |
| 34.6 | 80.4 | 0 | 1.67 | 0.26 | 1680 |
| 124.11 | 71 | 15 | 2.77 | 0.135 | 2160 |
| 100 | 42.19 | 0 | 1.52 | 0.23 | 1010 |
| 100 | 72.23 | 25 | 28.12 | 0.2 | 1160 |
| 400 | 75.5 | 25 | 28.12 | 0.2 | 12230 |
| 100 | 52.6 | 0 | 1.52 | 0.23 | 1520 |
| 100 | 58.7 | 15 | 12.78 | 0.215 | 1130 |
| 100 | 85.91 | 60 | 25.93 | 0.029 | 4300 |
| 82.74 | 57 | 0 | 1.88 | 0.148 | 2722 |
| 41.4 | 56 | 5 | 1.88 | 0.148 | 1355 |
| 124.11 | 65 | 45 | 9.19 | 0.0865 | 2049 |
| 124.11 | 55 | 5 | 1.88 | 0.148 | 2275 |
| 100 | 60.56 | 15 | 12.78 | 0.215 | 1930 |
| 29.8 | 72 | 0 | 2.27 | 0.15 | 930 |
| 46.2 | 61.7 | 0 | 2.27 | 0.15 | 1263 |
| 46.2 | 42.6 | 0 | 2.27 | 0.15 | 466 |
| 46.2 | 43.3 | 0 | 2.27 | 0.15 | 615 |
| 132.7 | 61.4 | 0 | 2.27 | 0.15 | 2119 |
| 124.11 | 71 | 5 | 1.88 | 0.148 | 3412 |
| 15.7 | 93 | 28 | 5.88 | 0.13 | 432 |
| 100 | 64.61 | 25 | 28.12 | 0.2 | 890 |
| 400 | 66.79 | 25 | 28.12 | 0.2 | 6770 |
| 41.4 | 54 | 35 | 4.33 | 0.107 | 789 |
| 100 | 73.14 | 25 | 28.12 | 0.2 | 1420 |
| 41.4 | 68 | 0 | 1.88 | 0.148 | 2281 |
| 160 | 43.3 | 0 | 2.27 | 0.15 | 2027.3 |
| 100 | 72.05 | 25 | 28.12 | 0.2 | 970 |
| 34.7 | 72.1 | 0 | 2.27 | 0.15 | 1085 |
| 100 | 70.24 | 25 | 28.12 | 0.2 | 1920 |
| 15.35 | 86.2 | 28 | 5.88 | 0.13 | 425 |
| 124.11 | 61 | 15 | 2.77 | 0.135 | 2078 |
| 80 | 42.4 | 0 | 2.27 | 0.15 | 997.5 |
| 100 | 81.77 | 0 | 1.52 | 0.23 | 5260 |
| 100 | 86.55 | 60 | 25.93 | 0.029 | 3500 |
| 41.4 | 71 | 45 | 9.19 | 0.0865 | 1920 |
| 124.11 | 69 | 45 | 9.19 | 0.0865 | 2908 |
| 160 | 61.7 | 0 | 2.27 | 0.15 | 10868 |
| 32.3 | 88.5 | 28 | 5.88 | 0.13 | 508 |
| 80 | 61.6 | 0 | 2.27 | 0.15 | 1730.9 |
| 41.4 | 64 | 35 | 4.33 | 0.107 | 809 |
| 100 | 9.38 | 0 | 1.52 | 0.23 | 670 |
| 82.74 | 72 | 35 | 4.33 | 0.107 | 1256 |
| 29.1 | 71.8 | 0 | 1.67 | 0.26 | 1120 |
| 82.74 | 58 | 0 | 1.88 | 0.148 | 2878 |
| 28.8 | 76.3 | 0 | 2.27 | 0.15 | 1405 |
| 100 | 90.18 | 60 | 25.93 | 0.029 | 3710 |
| 124.11 | 52 | 0 | 1.88 | 0.148 | 3391 |
| 41.4 | 60 | 0 | 1.88 | 0.148 | 1749 |
| 14.13 | 55 | 28 | 5.88 | 0.13 | 385 |
| 124.11 | 76 | 35 | 4.33 | 0.107 | 1680 |
| 82.74 | 78 | 0 | 1.88 | 0.148 | 5507 |
| 100 | 59.38 | 0 | 1.52 | 0.23 | 2980 |
| 400 | 71.87 | 25 | 28.12 | 0.2 | 5350 |
| 41.4 | 56 | 15 | 2.77 | 0.135 | 869 |

Table S-2. Testing data set

| Sigma c | Dr(%) | FC (%) | Cu | D50(mm) | W (J/m3) |
| --- | --- | --- | --- | --- | --- |
| 33.9 | 58.5 | 0 | 2.27 | 0.15 | 600 |
| 100 | 53.73 | 15 | 12.78 | 0.215 | 1350 |
| 141.3 | 71.07 | 0 | 2.27 | 0.15 | 6238 |
| 31.7 | 67.2 | 28 | 5.88 | 0.13 | 475 |
| 100 | 73.96 | 0 | 1.52 | 0.23 | 2660 |
| 400 | 73.96 | 0 | 1.52 | 0.23 | 15000 |
| 41.4 | 60 | 5 | 1.88 | 0.148 | 1609 |
| 100 | 70.78 | 25 | 28.12 | 0.2 | 780 |
| 28.7 | 60.7 | 0 | 2.27 | 0.15 | 590 |
| 124.11 | 47 | 45 | 9.19 | 0.0865 | 1713 |
| 41.4 | 77 | 35 | 4.33 | 0.107 | 894 |
| 100 | 62.07 | 25 | 28.12 | 0.2 | 740 |
| 100 | 88.02 | 0 | 1.52 | 0.23 | 6470 |
| 82.74 | 54 | 35 | 4.33 | 0.107 | 941 |
| 41.4 | 60 | 15 | 2.77 | 0.135 | 1139 |
| 82.74 | 72 | 5 | 1.88 | 0.148 | 2781 |
| 34.07 | 60.2 | 0 | 1.67 | 0.26 | 887 |
| 124.11 | 62 | 35 | 4.33 | 0.107 | 1352 |
| 41.4 | 70 | 5 | 1.88 | 0.148 | 1650 |
| 41.4 | 54 | 45 | 9.19 | 0.0865 | 921 |
| 28.4 | 64.7 | 0 | 2.27 | 0.15 | 785 |
| 82.74 | 55 | 5 | 1.88 | 0.148 | 1704 |
| 80 | 63 | 0 | 2.27 | 0.15 | 1829.1 |
| 100 | 25 | 0 | 1.52 | 0.23 | 810 |
| 97.2 | 62.1 | 0 | 2.27 | 0.15 | 3828 |
| 80 | 44.9 | 0 | 2.27 | 0.15 | 1162.1 |
| 400 | 48.44 | 0 | 1.52 | 0.23 | 4750 |
| 100 | 87.73 | 60 | 25.93 | 0.029 | 2680 |
| 31.5 | 72 | 28 | 5.88 | 0.13 | 502 |
| 100 | 71.51 | 25 | 28.12 | 0.2 | 1670 |
| 82.74 | 66 | 5 | 1.88 | 0.148 | 2517 |
| 89.6 | 44.9 | 0 | 2.27 | 0.15 | 711 |
| 46.7 | 63 | 0 | 2.27 | 0.15 | 537 |
| 40 | 41.6 | 0 | 2.27 | 0.15 | 398.2 |
| 74.5 | 62.7 | 0 | 2.27 | 0.15 | 831 |
| 124.11 | 62 | 15 | 2.77 | 0.135 | 2120 |
| 124.11 | 68 | 0 | 1.88 | 0.148 | 6257 |
| 82.74 | 54 | 45 | 9.19 | 0.0865 | 1305 |
| 82.74 | 55 | 15 | 2.77 | 0.135 | 1317 |
| 41.4 | 44 | 0 | 1.88 | 0.148 | 1687 |
| 100 | 64.43 | 25 | 28.12 | 0.2 | 590 |
| 100 | 25.52 | 0 | 1.52 | 0.23 | 730 |
| 82.74 | 60 | 45 | 9.19 | 0.0865 | 1749 |

Table S3- Hyperparameters of the applied models.

| Normalization mode | Model | Hyperparameters |
| --- | --- | --- |
| Linear | ANFIS-Sub | Maximum number of epochs = 200 |
|  |  | Error goal = 0 |
|  |  | Initial step size = 0.01 |
|  |  | Step size decrease rate =0.9 |
|  |  | Step size increase rate = 0.1 |
|  |  | Maximum number of epochs = 200 |
|  |  | Error goal = 0 |
|  | ANFIS-FCM | Initial step size = 0.01 |
|  |  | Step size decrease rate =0.09 |
|  |  | Step size increase rate = 0.1 |
|  |  | Number of Clusters =5 |
|  |  | Partition matrix exponent = 2 |
|  |  | Maximum number of iterations = 200 |
|  |  | Minimum Improvement = 1e-5 |
|  |  | Maximum number of epochs = 200 |
|  | ELM | Hidden nods = 10  Activation function = Log-sigmoid transfer function  Rangs of weights and biases are -1 to 1 |
|  | ELM-DOA | Hidden nods = 15 |
|  |  | Activation function = Log-sigmoid transfer function |
|  |  | Rangs of weights and biases are -1 to 1 |
|  |  | Population numbers = 31 |
|  |  | Maximum iteration = 150 |
| Nonlinear | ANFIS-Sub | Error goal = 0 |
|  |  | Initial step size = 0.01 |
|  |  | Step size decrease rate =0.9 |
|  |  | Step size increase rate = 1.1 |
|  |  | Maximum number of epochs = 200 |
|  |  | Error goal = 0 |
|  | ANFIS-FCM | Initial step size = 0.01 |
|  |  | Step size decrease rate =0. 9 |
|  |  | Step size increase rate = 1.1 |
|  |  | Number of Clusters =5 |
|  |  | Partition matrix exponent = 2 |
|  |  | Maximum number of iterations = 200 |
|  |  | Minimum Improvement = 1e-5 |
|  | ELM | Hidden nods = 33  Activation function = Log-sigmoid transfer function  Rangs of weights and biases are -1 to 1 |
|  | ELM-DOA | Hidden nods = 23  Activation function = Log-sigmoid transfer function  Rangs of weights and biases are -1 to 1  Population numbers = 31  Maximum iteration = 150 |
